# Biplane Reduction: A Novel Technique for Restoring Fibula Length in Maisonneuve Fracture

**DOI:** 10.1111/os.14130

**Published:** 2024-07-10

**Authors:** Qigang Zhong, Hu Yang, Chaoyue Huai, Yunfeng Yao, Yang Xie, Junfeng Zhan

**Affiliations:** ^1^ Department of Orthopedics Maanshan People's Hospital Maanshan China; ^2^ Department of Orthopedics The Second Affiliated Hospital of Anhui Medical University Hefei China; ^3^ Institute of Orthopedics, Research Center for Translational Medicine The Second Affiliated Hospital of Anhui Medical University Hefei China

**Keywords:** Fibula Length, Fibula Shortening, Maisonneuve Fracture, Reduction

## Abstract

**Objective:**

Fibular length is important for ankle stability. Failure to adequately restore and stabilize fibula length may result in malunion, hindering postoperative functional exercises and recovery of ankle function. However, there is no unified and effective reduction method for fibular shortening in Maisonneuve fractures. In this study, we introduce the biplane reduction as an effective surgical technique for restoring the length of the fibula in Maisonneuve fractures.

**Methods:**

This retrospective study enrolled 12 patients preoperatively diagnosed with Maisonneuve fractures between June 2019 and June 2022. All patients underwent our biplane reduction technique to restore the length of their fibula. Operation time, hospital stay, complications, FTA (the angle between the fibular tip and the superior articular surface of the talus), visual analog pain score, and the American Orthopaedic Foot and Ankle Society ankle‐hindfoot score were recorded. An independent sample t‐test was used for component comparisons, and a paired sample t‐test or one‐way analysis of variance was used for intra‐group comparisons.

**Results:**

No patient was lost to follow‐up and no obvious postoperative complications were observed. After operation, FTA recovered from 37.52 ± 0.37 ° to 40.42 ± 0.43 °, which was significantly improved compared with that before operation (*p* < 0.01). At the last follow‐up, both the VAS and AOFAS scores showed significant improvement compared to pre‐surgery scores.

**Conclusions:**

The reduction technique proposed in this study to restore the length of the fibula in Maisonneuve fractures is simple and effective, does not require special equipment, and has the advantage of directly examining the reduction condition.

## Graphical abstract

The anterolateral incision of the anterior edge of the fibula was made slightly arcually inward from approximately 8 cm above the level of the ankle joint to the level of the ankle joint. The skin and fascia were cut layer by layer. The extensor support band was cut open and the anterior tibial muscles were pulled medially to expose the inferior tibiofibular commissure and distal fibula. During the operation, the inferior tibiofibular syndesmosis was completely separated, and the interosseous membrane was torn. The inferior tibiofibular syndesmosis joint was fully cleaned, parallel to the coronal plane of the ankle joint, and a 2.0 mm Kirschner wire was inserted vertically on the tibial shaft 8 inches above the level of the ankle joint, 0.5–1.0 inches away from the anterior edge of the fibula, and approximately 7 feet above the level of the ankle joint, perpendicular to the coronal plane of the ankle joint, a common screw was placed vertically on the fracture piece of the distal fibula; the distance between the two was approximately 1.0 mm. A Kirschner needle retractor was placed between the Kirschner needle and screw, and a stretching technique was used to restore the length of the fibula. Taking the length of the contralateral fibula as a reference, intraoperative fluoroscopy showed that after the fibula length recovered well, a 2.0 mm Kirschner wire was used to temporarily fix the inferior tibiofibular syndesmosis, and a 3.5 mm metal cortical bone screw was inserted to fix the inferior tibiofibular syndesmosis at approximately 2.0 cm and 4.0 cm above the level of the ankle joint.

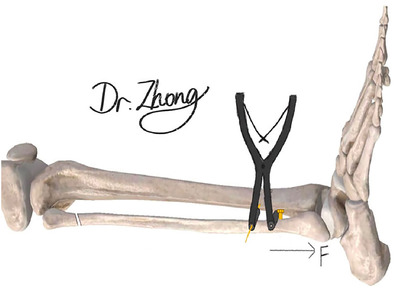

## Introduction

Maisonneuve fracture is a rare ankle fracture (accounting for approximately 7% of all ankle fractures) that can be classified into pronation and external rotation types in the Lauge‐Hansen classification and C3 type in the AO classification.^1^, ^2^ Owing to the difficulty in diagnosis and high misdiagnosis rate, approximately 20% of patients have a poor prognosis.^3^ A Maisonneuve fracture occurs when the ankle joint is subjected to external rotation violence, leading first to a medial ankle injury and resulting in a medial malleolus fracture or triangular ligament tears. The fibula is not only subjected to a pulling force of lateral rotation but also the impact force of the talus, leading to tearing of the interosseous membrane, injury to the inferior tibiofibular ligament, and high fibula fracture.^1^, ^3^, ^4^ Therefore, Maisonneuve fractures are theoretically unstable, and early surgical treatment is recommended.^3^

The surgical treatment of Maisonneuve fractures has three main purposes: (1) reduction and fixation of fibula fractures, (2) reduction and stabilization of medial malleolus fractures or repair of triangular ligament tears, and (3) treatment of inferior tibiofibular syndesmosis injury.^3^, ^5^, ^6^ Maisonneuve fractures can cause shortening and external rotation of the fibula. Studies have suggested that the increase in contact pressure of the tibiotalar joint is related to poor reduction of the fibula, and shortening by 2 mm will significantly increase the pressure, which is also a major risk factor for late revision surgery for ankle fracture.^3^, ^7^ Dattani et al. emphasized the importance of correcting the fibular length and external rotation in biomechanical studies. The outward movement of the talus by 1 mm reduced the tibial contact area by 42%.^8^ Reconstructing the tibiofibular space, restoring the length of the fibula, and stabilizing the position of the fibula in the tibial notch are important to maintain ankle stability, restore normal ankle biomechanics, and obtain a good prognosis. At present, restoration of the fibula length is limited to traction, Kirschner wire temporary fixation of both ends of the fracture, or the combined use of a distraction device and reduction forceps to correct the fibula length. Most of the aforementioned reduction methods have difficulty achieving these expectations, and shortening the fibula with a longer fracture line is even more difficult. Therefore, this study aimed to: (1) introduce the biplane reduction, an effective surgical technique for restoring the length of the fibula in Maisonneuve fractures; (2) evaluate the feasibility and safety of this reduction technique; and (3) compare the advantages and disadvantages of this reduction technique with those of traditional reduction. We hypothesize that biplane reduction is an effective surgical technique for recovery of fibular shortening in Maisonneuve fractures.

## Methods

### Inclusion and Exclusion Criteria

This study was approved by the local ethics committee (YX2023‐101), and all patients provided signed informed consent. The inclusion criteria were as follows: (1) Maisonneuve fracture was diagnosed using preoperative imaging data, (2) the interval between injury and admission was not more than 5 days, (3) the patients were aged 18–65 years, and (4) fibula length would be restored by the biplane reduction technique proposed in this study. The exclusion criteria comprised having: (1) an open fracture, (2) an old fracture of the ankle, (3) a dysfunctional ankle joint before injury, and (4) a bilateral primary ankle fracture.

### Patient Demographics

In this study, we included 12 patients with Maisonneuve bone fractures (four left and eight right fractures) between June 2019 and June 2022. The patients were eight males and four females (average age, 32.83 ± 11.46 years; average body mass index, 23.28 ± 3.97 kg/m^2^). The biplane reduction technique proposed in this study was used to restore the fibular length. Patient demographics, Danis–Weber type, injury causes, and operative therapy are summarized in Table 1.

**TABLE 1:** Patient demographics and causes of injury.

Patient ID	Age (years)	Sex	Side	BMI (kg/m^2^)	Causes of injury	Danis–Weber type	Operative therapy
1	19	M	R	21.36	Fall down the stairs	C	ORIF
2	46	M	R	25.44	Slipping on the ground	C	ORIF
3	25	F	R	24.32	Fall down the stairs	C	ORIF
4	41	M	L	23.93	Non‐motor vehicle accident	C	ORIF
5	36	F	L	17.44	Non‐motor vehicle accident	C	ORIF
6	22	M	R	17.36	Fall down the stairs	C	ORIF
7	20	M	L	24.13	Tripping on the ground	C	ORIF
8	30	M	R	32.24	Slipping on the ground	C	ORIF
9	32	F	R	24.74	Non‐motor vehicle accident	C	ORIF
10	41	F	L	24.14	Sprain	C	ORIF
11	56	M	R	24.02	Slipping on the ground	C	ORIF
12	26	M	R	20.19	Non‐motor vehicle accident	C	ORIF

Abbreviations: BMI, body mass index; F, female; L, left; M, male; ORIF, open reduction and internal fixation; R, right.

### Operation Procedure

#### Anesthesia and Position

All surgeries were performed by the same group of doctors. All patients were placed in a horizontal position, administered epidural or general anesthesia, and routinely disinfected with towels using a tourniquet.

### Surgical Technique

#### Preoperative Preparation

Radiographic data of the contralateral ankle joint were obtained in the operating room before the operation. These data was used as a standard for evaluating the length and rotation of the normal fibula and the recovery of the fibula length of the affected limb.

#### Surgical Approach and Exposure

The surgical approach involved an anterolateral fibular incision combined with a standard medial malleolar incision. The anterolateral incision of the anterior edge of the fibula was made slightly arcually inward from approximately 8 cm above the level of the ankle joint to the level of the ankle joint. The skin and fascia were cut layer by layer. The extensor support band was cut open, and the anterior tibial muscles were pulled medially to expose the inferior tibiofibular commissure and distal fibula. During the operation, the inferior tibiofibular syndesmosis was completely separated, and the interosseous membrane was torn. To facilitate its anatomical reduction, the lower tibiofibular joint was thoroughly cleaned to avoid being compressed in the space.

#### Reset Technique

**FIGURE 1:**
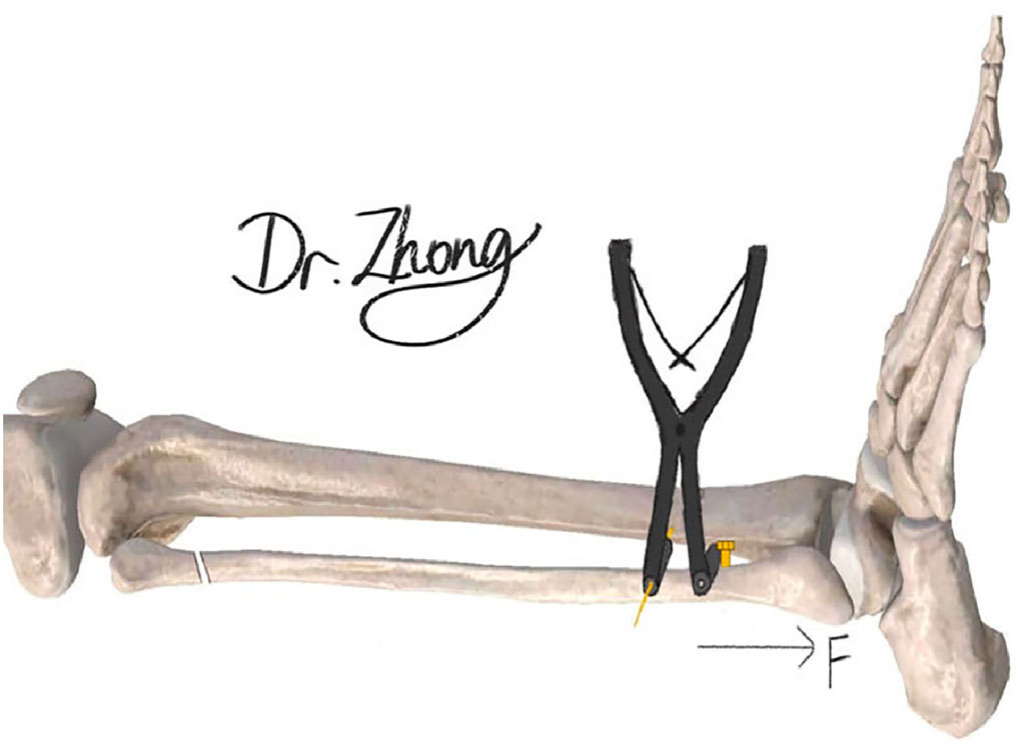
Diagram of biplane reduction for restoring fibula length in Maisonneuve fracture.

Then, fibula length was restored by the method proposed in this study and the schematic diagram of this method is shown in Figure 1. Firstly, parallel to the coronal plane of the ankle joint, 8 cm above the level of the ankle joint, and approximately 0.5–1.0 cm from the leading edge of the fibula for the insertion point, a 2.0 mm diameter Kirschner needle was vertically driven into the tibial shaft. The purpose is to act as a side resistance during reducing. Secondly, approximately 7 cm above the level of the ankle joint, perpendicular to the coronal plane of the ankle joint, a common screw was placed vertically on the fracture piece of the distal fibula; the distance between the two implants was approximately 1.0 cm. In order to restore the fibula length, we placed the Kirschner spreader between the Kirschner needle and the screw. Subsequently, one end of the spreader was inserted into the Kirschner needle, and the other end was lodged between the fibula and the tail cap of the screw. Using the end of the Kirschner wire as a barrier, the spreader was forcefully pushed open, pulling the long axis of the fibula to restore its length. When a distal fibula rotation deformity was present, a towel clamp was used to clamp the distal fibula and the anterior medial tibia to correct the external rotation deformity.

#### Fixation after Reduction

**FIGURE 2:**
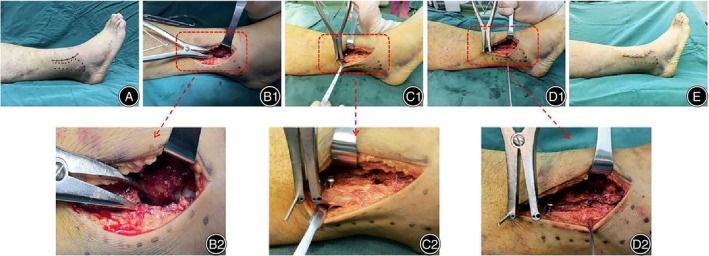
A 46‐year‐old male had a Maisonneuve fracture with pain and swelling of the right ankle and tenderness of the proximal fibula. The biplane reduction technique was used to restore the length of the fibula. (A) Anterolateral incisions were designed preoperatively. (B) Separation of inferior tibiofibular syndesmosis and tearing of interosseous membrane were observed during the operation. (C) A 2.0 mm Kirschner wire was placed perpendicular to the tibial shaft and then perpendicular to the coronal plane of the ankle joint. A common screw was placed vertically on the fracture block of the distal fibula, the distance between the two was approximately 1.0 mm. A Kirschner pin was placed between the Kirschner wire and the screw. (D) Using the end of the Kirschner needle as resistance, a pull force along the long axis to the distal end of the fibula was applied to restore the length of the fibula. (E) Postoperative irrigation and suture wound.

**FIGURE 3:**
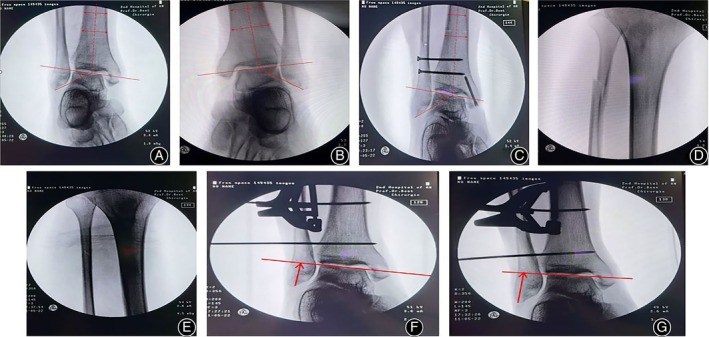
Intraoperative impact data and measurement of the FTA† angle. (A) Preoperative X‐ray of the affected side. (B) Preoperative X‐ray of the healthy side. (C) Postoperative X‐ray of the affected side. (D) Before the fracture of the proximal fibula was reduced. (E) After the reduction of the proximal fibular fracture. (F) Before the resetting device is assembled and sputtered. (G) The reset device is assembled and stretched out. † FTA, the angle between the intersection of the tibial dissection axis and the superior articular surface of the talus and the angle formed by the superior articular surface of the talus.

Taking the length of the contralateral fibula as a reference, intraoperative fluoroscopy showed that after the fibula length was well restored, a 2.0 mm Kirschner wire was used to temporarily fix the inferior tibiofibular syndesmosis, and a 3.5 mm metal cortical bone screw was inserted to fix the inferior tibiofibular syndesmosis at approximately 2.0 cm and 4.0 cm above the level of the ankle joint. The medial and posterior malleoli were treated routinely. Fluoroscopy showed good reduction of the fracture, and the wound was washed and sutured layer‐by‐layer after proper internal fixation. A typical example is shown in Figure 2. Intraoperative imaging data are shown in Figure 3.

### Postoperative Care and Rehabilitation

After the operation, patients were routinely administered symptomatic treatments, such as anti‐inflammatory drugs, pain relief, and swelling reduction. All patients were fixed with a short‐leg plaster for 2 weeks, and active and passive non‐weight‐bearing ankle exercises began 2 weeks later. The stitches were removed 2 weeks after the operation, and weight was gradually loaded 8 weeks after the operation until complete weight bearing.

### Observation Assessment

The perioperative data of all patients was recorded, including the operation time, hospital stay, and average follow‐up time. Complications in all patients during the follow‐up period were recorded, including incision infection, failure or loosening of internal fixation, failure to restore fibula length during the operation, and deep venous thrombosis of the lower extremities. The angle between the fibular tip and the superior articular surface of the talus, referred to as the FTA, was measured using radiographic imaging before and after the reduction of the affected limb, and contralateral radiographs before and after the operation.^9^ This angle, formed by the intersection of the tibial dissection axis and the superior articular surface of the talus and the angle formed by the superior articular surface of the talus, served to evaluate the severity of fibula shortening and assess the recovery of fibula length. The measurement method is shown in Figure 3. A clinical evaluation was performed both before and during follow‐up, including the visual analog pain score (visual analog scale, VAS) and the American Orthopaedic Foot and Ankle Society (AOFAS) ankle‐hindfoot score. The VAS score ranged from 0 to 10, and the AOFAS score ranged from 0 to 100, with ratings of 90–100 considered excellent, 75–89 as good, 50–74 as average, and <50 as poor.

### Statistical Analysis

Statistical analyses were performed using SPSS (version 26.0; IBM Corp. Released 2019. IBM SPSS Statistics for Windows, Chicago, IL, USA). The measurement data were expressed as the mean ± standard deviation. Paired sample t‐tests or single factor analyses of variance were performed to compare the VAS and AOFAS scores at different time points, and the independent sample t‐test was performed to compare the FTA between groups. *P*‐values of less than 0.05 indicated statistical significance.

## Results

### General Results

No patient was lost to follow‐up. The average follow‐up time, operation time, and hospital stay were 22.72 ± 3.16 months, 89.58 ± 19.73 minutes, and 11.92 ± 3.50 days, respectively. In all patients, this method effectively restored the length of the fibula, after maintenance by rigid fixation.

### Complications

There was no case of iatrogenic fracture caused by an invasive operation, and no undercorrection or overcorrection was observed on radiography after surgery. During follow‐up, there was a case of a broken lower tibiofibular screw in a patient; the broken part of the lower tibiofibular screw was not removed during the internal fixation removal operation with patient's informed consent. The wounds of all patients healed in one stage, and there were no complications, such as superficial or deep infections, deep venous thrombosis of the lower extremities, or loosening and failure of internal fixation.

### Fibula Length Recovery

According to the patient imaging data, before the operation the FTA of the healthy side was 40.72 ± 0.57 ° and that of the affected side was 37.52 ± 0.37 °. The difference between both FTAs was statistically significant (*p* < 0.01), indicating that the fibula length of the affected limb was significantly shortened. After the fibula length was restored, the FTA of the affected side was 40.42 ± 0.43 °, which was not statistically significant compared with that of the healthy side (*p* < 0.01), indicating that the fibula length had significantly improved.

### Clinical Evaluation

The mean preoperative VAS score among patients was 6.83 ± 1.40, and the mean AOFAS score was 41.00 ± 5.05. Subsequently, both VAS and AOFAS scores exhibit significant increases (*p* < 0.01). During the final follow‐up, the VAS and AOFAS scores were 0.91 ± 0.54 and 95.18 ± 4.22, respectively. Eleven cases were rated as excellent and one as good, with the excellent and good rates being up to 100%. Table 2 presents the VAS and AOFAS scores at different follow‐up intervals.

**TABLE 2:** Sample follow‐up characteristics of the patients.

	Pre‐operation	1 month after surgery	3 months after surgery	6 months after surgery	1 year after surgery	Last follow‐up	F‐value	*p*‐value
AOFAS	41.00 ± 5.05	61.67 ± 7.56	78.33 ± 7.08	86.08 ± 6.92	94.58 ± 4.32	95.18 ± 4.22	1426.09	<0.001
VAS	6.83 ± 1.40	4.08 ± 1.38	1.67 ± 1.07	1.25 ± 0.75	0.92 ± 0.67	0.91 ± 0.54	324.55	<0.001

Abbreviations: AOFAS, American Orthopaedic Foot and Ankle Society ankle‐hindfoot score; VAS, visual analog scale.

## Discussion

### Current Findings

In our series of studies, we have determined that the biplane reduction technique is both safe and effective for restoring the length of the fibula in Maisonneuve fractures. To our knowledge, we are the first to propose this method to restore the fibula length in Maisonneuve fractures. In this study, the length of the fibula was successfully restored, and rigid fixation was performed in 12 patients. This method is simple, effective, and does not require special instrumentation, offering the advantage of restoring both the physiological and anatomical positions of the fibula.

### Comparison with Other Techniques for Restoring Fibula Length

In the past, fibular fractures received little attention, as the fibula was primarily considered as the attachment point of muscles and ligaments, or even as a bone graft source.^10^ However, clinical research in recent years has emphasized the importance of fibular length for ankle stability, biomechanical research, and medium‐ and long‐term follow‐up.^3^, ^8^, ^11^, ^12^ Proximal fibular fractures in Maisonneuve fractures are usually not directly reduced and fixed.^13^ Some scholars have described various techniques for restoring the length of the fibula; however, most of them are limited to osteotomy and orthopaedic surgery, unsuitable for traumatic injuries. Riedel et al. partially restored the length of the fibula using two suture button devices combined with screws. However, this type of suture button device is not popular and difficult to use.^6^ Huang et al. proposed the use of a slender combined hole of the locking compression plate to prolong the length of the fibula,^14^ which avoids extending the distal end of the lateral incision. Nonetheless, the thickness of the composite plate leads to a protuberance in the epidermis, hindering incision healing and functional exercise, imposing economic burden on patients. Fitzpatrick described a technique to prevent shortening of the fibula by holding the distal fibula with reduction clamps, providing continuous traction, and temporarily fixing it with a Kirschner wire when the muscle and surrounding soft tissue are relaxed and the length of the fibula is satisfactory.^15^ However, uncertainties are associated with this method, including shortening of the fibula, swelling of local tissue, physical strength of the patients, and need for revision surgery. Micheal et al. introduced a distraction technique similar to this method in the case of nonunion of a fibular fracture. Screws were fixed to the tibia and fibula in two planes, and the length of the fibula was restored using a lamina retractor.^16^ However, this distraction technique is unstable, and it is difficult to obtain intraoperative fluoroscopy without maintenance. It also carries the risk of fracture because of the uneven force of the fibula screw.

### Technical Characteristic

The conventional use of Kirschner's needle spatters is limited to both ends of the fracture line. However, in Maisonneuve fractures, the fibula fracture line occurs at the proximal end of the fibula. The method we propose can reduce the fibula fracture without the need for a proximal incision. Coronal and sagittal screws were inserted parallel to the ankle joint. Using the end of the Kirschner wire as resistance, a traction force parallel to the long axis is applied to the fibula to reduce its length. This reduction technique is stable, with one end of the Krantler needle spanner threaded into the Krantler needle fixed to the tibia and the other end lodged just between the fibula and the tail cap of the ordinary screw. Reconstructing the length of the fibula can be obtained directly through fluoroscopy without maintenance, and it is easy to adjust compared to the healthy side. When external rotation of the fibula is present, the deformity can be corrected by clamping the distal end of the fibula and the anterior medial end of the tibia with towel forceps.

### Advantages of Biplane Reduction Technique

The biplane reduction technique described in this paper prevents fibular shortening in Maisonneuve fractures. A Kirschner wire fixed at the end of the tibia was used for resistance, and a Kirschner pin opener was used to indirectly restore the length of the fibula. The operation is simple and reproducible without special instruments, and there is less damage to the soft tissue of the incision, significantly preventing injury to the common and superficial peroneal nerves.^17^

### Feasibility and Safety

In this study, the FTA of the affected side recovered from 37.52 ± 0.37 ° to 40.42 ± 0.43 °, and there was no significant difference between the affected side and the healthy side, indicating that the proposed method could effectively restore the length of the fibula. In addition, both the Kirschner wire at the end of the tibia and the screw at the end of the fibula were subjected to a uniform force, which avoided the problem of fracture to a significant extent. Restoring the normal physiological and anatomical position of the fibula is beneficial for postoperative functional exercises and restoring the function of the ankle joint. At the last follow‐up, one of the 12 patients had a good result; the other 11 had excellent results. The AOFAS score was 94.58 ± 4.32, which was satisfactory.

### Limitations and Strengths

This study has the following limitations. First, due to the low incidence of Maisonneuve fractures, the sample size of this study was small and did not cover all degrees of Maisonneuve fractures, especially the severity of fibular shortening and external rotation. Second, this study did not compare with other treatments, thus lacking evidence compared with prospective studies. Besides, there are some potential problems that may exist in clinical application. There is a risk of iatrogenic fractures and overcorrection. When we place the screw, we should pay attention to select a place with more bone to avoid iatrogenic fracture of the fibula, it is recommended to obtain the length recovery of the fibula under X‐ray after we have spread the Kirschner spreader. However, for Maisonneuve fractures, the traditional reduction square method may lead to loss of length after reduction, and the long‐term effects cannot be guaranteed. Our novel biplane reduction technique for restoring fibular length in Maisonneuve fractures is the first of its kind. In our current follow‐up, the effect was accurate, and the prognosis was good. The distance between the screw and Kirschner wire can be used directly to achieve fibula length recovery, which avoids cases of insufficient reduction and overcorrection, and provides a new surgical technique for foot and ankle surgeons. This paper provides a simple surgical technique that has advantages in restoring fibula length and maintaining normal anatomical characteristics of the ankle points.

## Conclusions

Biplane reduction is an effective surgical technique for recovery of fibular shortening in Maisonneuve fractures. It is safe, simple, effective, does not require special equipment, can be used to directly set the degree of reset, and is easy to maintain, with advantages over existing reduction technology.

## Conflict of Interest Statement

The authors declare that they have no competing interests.

## Author Contributions

Junfeng Zhan and Yang Xie developed the idea of the study, Qigang Zhong participated in its design and helped to draft the manuscript. All authors read and approved the final manuscript. Qigang Zhong, Hu Yang, and Chaoyue Huai contributed equally to this work.
